# Chemsex Practices and Health-Related Quality of Life in Spanish Men with HIV Who Have Sex with Men

**DOI:** 10.3390/jcm10081662

**Published:** 2021-04-13

**Authors:** Nicolás Ruiz-Robledillo, Rosario Ferrer-Cascales, Irene Portilla-Tamarit, Cristian Alcocer-Bruno, Violeta Clement-Carbonell, Joaquín Portilla

**Affiliations:** 1 Department of Health Psychology, University of Alicante, 03690 Alicante, Spain; nicolas.ruiz@ua.es (N.R.-R.); rosario.ferrer@ua.es (R.F.-C.); cristian.albru@ua.es (C.A.-B.); violeta.clement@ua.es (V.C.-C.); 2 Alicante Institute for Health and Biomedical Research (ISABIAL–FISABIO Foundation), 03010 Alicante, Spain; portilla_joa@gva.es; 3 Department of Infectious Diseases, General University Hospital of Alicante, 03010 Alicante, Spain; 4 Spanish Network of Excellence on HIV Research, RIS, 28029 Madrid, Spain; 5 Department of Clinical Medicine, Miguel Hernández University, 03016 Alicante, Spain

**Keywords:** Chemsex, HIV, men who have sex with men, health-related quality of life

## Abstract

Chemsex, a new risky sexual behavior involving participation in sexual relations under the influence of drugs, has shown a significantly increased prevalence in recent years. This fact entails a serious public health issue, especially when Chemsex is practiced by individuals with an HIV (Human Immunodeficiency Virus) diagnosis. Hence, analyzing the characteristics of Chemsex practices, associated sexual practices and the health outcomes of individuals who participate in Chemsex, is extremely important. The main aim of the present study is to analyze the prevalence and characteristics of the practice of Chemsex in a sample of 101 men with HIV who have sex with men who attended the Department of Infectious Diseases of the General University Hospital of Alicante (Spain). Furthermore, the association between Chemsex and Health-Related Quality of Life (HRQoL) was also assessed. Chemsex and sexual practices were evaluated by employing a questionnaire applied on an ad hoc basis. HRQoL was assessed by employing the Medical Outcomes Study HIV Health Survey (MOS-HIV). In total, 40.6% of the participants had practiced Chemsex during the last year. When sexual practices were compared between those individuals who practiced Chemsex and those who did not, the former presented a higher level of risky sexual behaviors, especially with occasional and multiple sexual partners. Regarding HRQoL, those individuals who practiced Chemsex exhibited a poorer HRQoL in the majority of domains, especially those participants who practiced it with a higher intensity. The present study points out the high prevalence of Chemsex practice between men with HIV who have sex with men in Spain. Moreover, this study highlights the negative effects of Chemsex on HRQoL, probably due to the mixed effects of higher levels of risky sexual practices and the consequences of drug consumption.

## 1. Introduction

Prevalence of HIV infection is a global public health issue—it is estimated that 1.7 million people were infected in 2018 alone [1]. In Spain, according to the annual report on the epidemiology of HIV, issued by the Ministry of Health, during 2018, 3244 new HIV diagnoses were reported, of which 85.3% were men. Of that percentage, 56.4% corresponded to men who have sex with men (MSM), an epidemiological term used to describe men who have sex with other men regardless of their sexual orientation or gender identity [2]. This trend in the data remains consistent among other Western countries [3] and represents a 19-fold increase in the probability of HIV infection [4].

These data confirm that HIV infection continues to be a highly prevalent public health concern, especially in the MSM population [5]. Although new strategies such as pre-exposure prophylaxis (PReP) have emerged [6,7], prevention through condoms remains the most recommended method due to its accessibility and effectiveness [8,9]. However, the lack of condom use as a prophylactic method against HIV has contributed to an increase in the transmission of HIV and other sexually transmitted diseases (STDs), such as syphilis, gonorrhea, hepatitis, urethritis, genital warts and chlamydia in this population [10].

Beyond the classical prophylactic methods, the availability of effective antiretroviral treatments has allowed people with HIV that have achieved and maintained an undetectable viral load not to sexually transmit the virus to others [11,12]. This fact, although it has allowed for great advances and a reduction in HIV transmission, has developed a false sense of security in people living with HIV (PLWHIV), leading to a significant decrease in the employment of prophylactic measures in their sexual relationships, resulting in increased risky sexual behaviors and transmission of other STDs [13,14,15].

Although it is known that the previously explained phenomena could be the basis for the risk of transmission of HIV and other STDs in PLWHIV, in recent years, it has become clear that there is a need to identify other risky sexual behaviors that lead to a high probability of transmission of the virus. However, most studies focused on promoting the use of condoms and identifying the factors promoting their use, as shown in the review conducted by Evans [16]. Nevertheless, there is little research on newer sexual phenomena such as Chemsex. This term refers to the development of risky sexual relations under the effects of drug use, with different purposes, such as increasing pleasure or prolonging the duration of sexual intercourse [17,18,19]. This type of risky sexual behavior, which is highly prevalent among MSM, in which substance use is mixed with the absence of prophylactic measures, carries a high risk of contagion, and therefore is extremely serious, not only because of the risk it poses to the individual himself, but to public health in general [20]. Drugs frequently used to increase sexual experiences include methamphetamine, crystal meth, mephedrone, and gamma-hydroxybutyrate (GHB) [20,21,22,23]. Although the prevalence of Chemsex is difficult to estimate because few individuals report the practice of such behaviors [23,24], previous studies estimate that about 30–45% of MSM have practiced Chemsex on at least one occasion [18,22,25]. This is why the identification of explanatory variables of these types of phenomena and their relationship with both the physical and psychological health of people is a challenge that should be taken up urgently by the scientific community. In spite of this, information on this subject is scarce, partly due to the novelty of the phenomenon and the difficulty in evaluating it.

Beyond the consequences of the practice of Chemsex increasing the risk of transmission of HIV and other STDs, it is important to know the consequences of this risky sexual practice on the health of the individuals who practice it. To the best of our knowledge, no previous studies have identified the effects of Chemsex on specific health markers, such as Health-Related Quality of Life (HRQoL). HRQoL is defined as a subjective outcome measure that assesses the influence of health status and physical, mental, and social functioning in relation to an individual’s goals [26,27,28] and is considered a measure of health outcomes and treatment adherence among people with HIV [29]. Although, as has been indicated, no previous studies have analyzed the relationship between Chemsex and HRQoL in men with HIV who have sex with men, it has been demonstrated that there is a negative association between some risky sexual behaviors, such as unprotected anal penetration and HRQoL in this population [30,31,32]. There are some emerging mechanisms in the recent literature that could help to explain the possible negative effects of Chemsex on the health of this population. In this sense, the practice of Chemsex is associated with a decreased treatment adherence in PLWHIV, limiting the effects of such treatments [33,34]. On the other hand, a review conducted by Degroote, Vogelaers, and Vandijck [35] associated drug use with poorer physical and mental health, finding that 25% of MSM with HIV who consume drugs reported negative effects on their lives [36], a decrease in their autonomy in activities of daily living [37], and a negative impact on their social and work relationships [38,39]. Mental health is also negatively affected among HIV-positive MSM who practice Chemsex, with 15% of MSM experiencing a negative impact on their mental health [40,41,42,43]. It is likely that the mixed effects of higher risky sexual practices and drug consumption consequences could be the basis of the health deterioration of this population. However, more studies are needed to characterize Chemsex practices and their consequences for HRQOL in men with HIV who have sex with men.

With all this in mind, the main aim of the present study was to characterize the phenomenon of Chemsex among a sample of Spanish men with HIV infection who have sex with men, and to analyze the relationship between Chemsex and HRQoL in this population.

## 2. Materials and Methods

### 2.1. Study Design and Participants

In this cross-sectional observational study, we included 101 men with HIV infection who have sex with men. The majority of them had undertaken advanced studies, were single, employed, with a mean economic income above 1500 euros per month, and self-identified as homosexuals. The characteristics of the participants are summarized in Table 1.

### 2.2. Variables

Chemsex practices were evaluated through an ad hoc questionnaire developed for this study. The questionnaire included questions regarding the participants’ Chemsex practices and their frequency during the last year, individuals with whom the participants engaged in Chemsex (stable sexual partner, occasional sexual partner and/or both), the type and the frequency of drugs consumed, and the time at which sex developed into Chemsex (e.g., whether drugs were taken before sex, during sex or before and during sex). The assessment of other sexual practices included questions regarding the frequency of specific sexual practices (double receptive anal penetration, double insertive anal penetration, receptive anal penetration, insertive anal penetration, fist penetration (fisting), anilingus, oral sex (fellatio) or mutual masturbation), and condom use (frequency of condom use, condom use during the last anal penetration or condomless anal intercourse at least once). These questions were answered by participants separately regarding stable and occasional sexual partners.

Sexually transmitted diseases (STDs). A single question was included to evaluate if participants were diagnosed with any of the following STDs at least once: genital warts, genital ulcers, urethritis, proctitis, syphilis, chlamydia, candidiasis or gonorrhea.

Health-Related Quality of Life (HRQoL). For the evaluation of HRQoL, we employed the Spanish version of the Medical Outcome Study-HIV Health Survey [44]. This questionnaire includes 11 subscales of HRQoL: General Health Perceptions (5 items), Pain (2 items), Physical Functioning (6 items), Role Functioning (2 items), Social Functioning (1 item), Mental Health (5 items), Energy/Fatigue (4 items), Cognitive Functioning (4 items), Health Distress (4 items), Quality of Life (1 item) and Health Transition (1 item). The scores obtained for these subscales can be quantified through the calculation of two general indexes: Physical Health Summary (PHS) and Mental Health Summary (MHS). Questions refer to the last two weeks and are rated on 2, 3, 5, and 6-point scales with a final score reflected on a scale from 0 to 100. Higher scores indicate greater health [45]. A recent reliability generalization meta-analysis pointed out that this instrument is highly reliable for the evaluation of HRQoL, with an average α coefficient for the total score of MOS-HIV of 0.91 and above 0.80 for all of the subscales, except for Role Functioning [46].

### 2.3. Procedure

The research was conducted in the Infectious Diseases Unit of the General University Hospital of Alicante in Spain. All patients in usual care between February 2020 and December 2020 who met the inclusion criteria were invited to participate in the study by filling in the indicated self-reported questionnaires. The confidentiality and anonymity of the obtained results was assured to participants throughout the whole study. Hence, to protect the confidentiality and anonymity of the data, codes were assigned to identify the participants. Furthermore, the research was conducted following the guidelines of the Declaration of Helsinki and the European Union Good Clinical Practice Standards, and the study was approved (26 February 2020) by the Ethics Committee of the General University Hospital of Alicante (PI2019/083). Inclusion criteria were: (1) HIV infection diagnosis, (2) ≥18 years-old, (3) being men who have sex with men, (4) being a patient receiving antiretroviral therapy and (5) having signed the informed consent to participate in the study. Exclusion criteria included: (1) the presence of comorbidities identified in medical records, dementia or other central nervous system diseases, mental health condition(s) diagnosis, viral chronic hepatitis, active cancer or infection, diabetes mellitus, high blood pressure, cardiovascular disease, hypothyroidism, malnutrition and other severe health conditions; (2) mental or physical impairments that could hinder participants’ ability to complete or understand the study questionnaires. After potential participants signed the informed consent, researchers exhaustively revised their medical records in order to identify any of the previously indicated exclusion criteria. Compliance with any of these exclusion criteria by patients led to exclusion from participation in the study. Participants were retained in the final sample only if they responded to all the questions involving the dependent variables.

### 2.4. Data Analysis

Descriptive analyses of the sociodemographic characteristics of the sample were carried out. The frequencies of characteristics of Chemsex and other sexual practices were calculated. Differences between participants who practiced Chemsex and those who did not in terms of STD diagnosis and condom use were analyzed by employing the chi-square statistic. Differences in the frequency of sexual practices and HRQoL between individuals who practice and do not practice Chemsex were identified through T-test analyses. Moreover, specific differences between Chemsex practitioners, depending on the time of the practice (e.g., whether drugs were taken before sex, during sex or before and during sex), were evaluated by employing non-parametric analyses in the form of the Kruskal–Wallis test. *p* < 0.05 was considered significant in all cases. All statistical analyses were conducted using SPSS version 24.0 (Armonk, NY, USA).

## 3. Results

### 3.1. Characteristics of Chemsex Practice in the Sample

Forty-one (40.6%) participants indicated that they participated in Chemsex during the last year and 60 (59.4%) indicated that they did not. Of these 41 participants, 8 (19.5%) had a stable partner, 19 (46.3%) had occasional sexual partners and 14 (34.2%) had both. With regard to the frequency of Chemsex, 20 (48.8%) participated in this sexual practice rarely, 15 (36.6%) sometimes, 5 (12.2%) very often and 1 (2.4%) always or almost always. Concerning the time at which participants consumed substances related to sexual practices, 10 (24.4%) consumed them before sex, 13 (31.6%) during sex and 18 (44%) before and during sexual practices. Table 2 and Table 3 include information regarding the time at which participants consumed each type of substance (never, before, during or before and during sexual practices) and the frequency of consumption of each type of substance.

### 3.2. Sexual Practices of Participants with Stable Sexual Partners (n = 59) and Differences Based on Chemsex Practice

**Figure 1:**
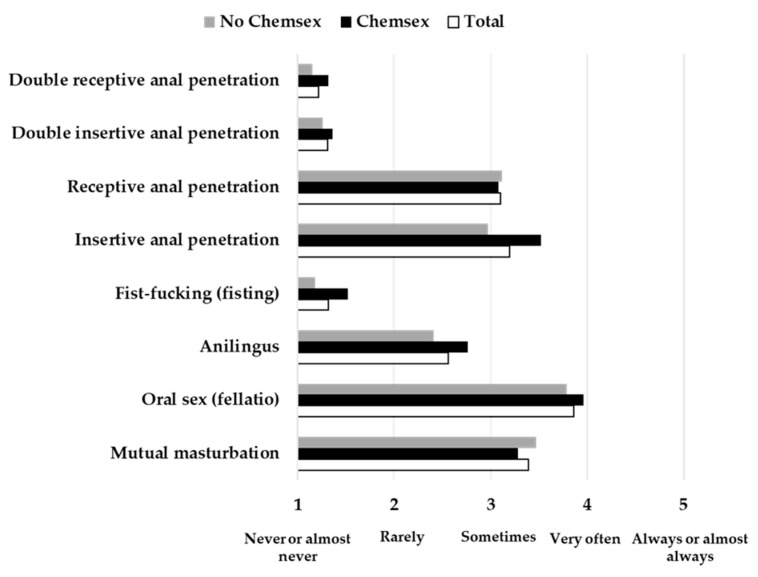
Frequency of sexual practices for all participants and for practitioners and non-practitioners of Chemsex separately, with stable sexual partners.

In the case of participants with stable sexual partners, 25 (42.4%) practiced Chemsex with their stable sexual partners and 34 (57.6%) did not. No differences were found between groups of Chemsex practitioners in terms of the frequency of any of the sexual practices evaluated with stable sexual partners (*p* > 0.05) (Figure 1).

### 3.3. Sexual Practices of Participants with Occasional Sexual Partners (n = 68) and Differences Based on Chemsex Practice

**Figure 2:**
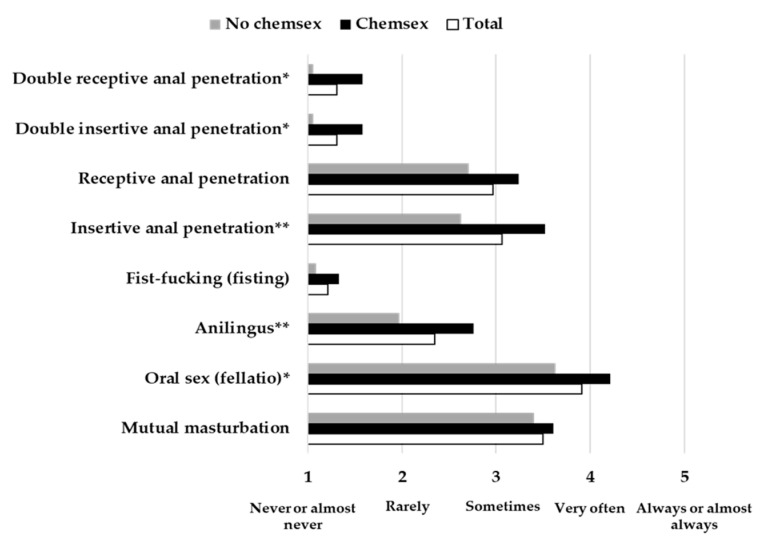
Frequency of sexual practices for all participants and for practitioners and non-practitioners of Chemsex separately, with occasional sexual partners. * *p* < 0.05, ** *p* < 0.01.

For participants with occasional sexual partners, 33 (48.5%) practiced Chemsex with occasional sexual partners and 35 (51.5%) did not. In this case, significant differences were found for double receptive anal penetration t (36.991) = −2.445, *p* = 0.019, *d* = 0.80; double insertive anal penetration t(37.226) = −2.498, *p* = 0.017, *d* = 0.81; insertive anal penetration t(66) = −2.992, *p* = 0.004, *d* = 0.73; anilingus t(66) = −2.821, *p* = 0.006, *d* = 0.69; and oral sex t(59.053) = −2.403, *p* = 0.019, *d* = 0.62. In all cases, those participants who practiced Chemsex exhibited a higher frequency of the development of these sexual practices with occasional sexual partners (Figure 2).

### 3.4. Differences in Condom Use between Participants Who Practiced Chemsex and Those Who Did Not

**Figure 3:**
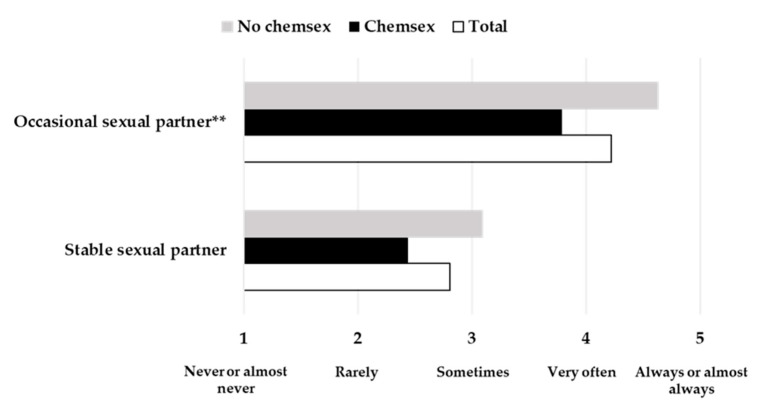
Frequency of condom use for all participants and for practitioners and non-practitioners of Chemsex separately, with occasional and stable sexual partners. ** *p* < 0.01.

Regarding the frequency of condom use, differences were found in its employment with occasional sexual partners between participants who practiced Chemsex and those who did not (t (43.607) = 3.053, *p* = 0.004, *d* = 0.92). In this case, participants who practiced Chemsex employed condoms with a lesser frequency with occasional sexual partners in comparison to their counterparts who did not practice Chemsex. No differences were found in the case of condom use with stable sexual partners (*p* > 0.05) (Figure 3).

As can be observed in Table 4, regarding condom use during the last anal penetration, differences were found between groups of participants based on the practice of Chemsex, specifically regarding the last anal penetration when this occurred with occasional sexual partners. In this sense, individuals who practiced Chemsex presented a lower use of condoms in the last anal penetration when this occurred with occasional sexual partners in comparison to individuals who did not practice Chemsex. No differences were found in the case of the last anal penetration when this occurred with a stable sexual partner (Table 4).

Regarding condomless anal intercourse at least once with stable and occasional sexual partners of discordant/unknown serological status, participants who practiced Chemsex exhibited a higher frequency of this practice with stable sexual partners. No differences were found regarding occasional sexual partners (Table 5).

### 3.5. Differences in Sexually Transmitted Diseases (STDs) between Participants Who Practiced Chemsex and Those Who Did Not

In the case of STD diagnosis in the evaluated sample, differences were found in the case of genital warts and urethritis. In both cases, participants who practiced Chemsex exhibited a higher prevalence of diagnosis of these diseases compared to their counterparts who did not practice Chemsex (Table 6).

### 3.6. Differences between Participants Who Practiced Chemsex and Those Who Did Not in Terms of HRQoL

Differences in HRQoL between participants based on Chemsex practice were assessed. As can be observed in Table 7, significant differences were identified in the following domains: General Health Perception, Pain, Energy/Fatigue, Mental Health, Cognitive Functioning, Physical Health Summary and Mental Health Summary. In all cases, participants who practiced Chemsex obtained lower scores in these dimensions, indicating a poor HRQoL.

### 3.7. Differences in HRQoL Depending on the Moment of the Practice of Chemsex Regarding Sex

The Kruskal–Wallis test was conducted to examine the differences in HRQoL according to the point at which sex became Chemsex (e.g., whether drugs were taken before sex, during sex or before and during sex). Significant differences were found in terms of Energy/Fatigue, Cognitive Functioning and Mental Health Summary. Post-hoc analyses were conducted, adjusting for significance (Bonferroni adjustment). In the case of Energy/Fatigue, no significant differences were found between specific groups. For Cognitive Functioning, significant differences were found between groups of participants who took drugs before sex and those who took drugs before and during sex (*p* = 0.004). Similarly, significant differences were found between the same groups in terms of their Mental Health Summary (*p* = 0.023). In both cases, participants who took drugs before and during sex presented lower scores in these dimensions of HRQoL in comparison to those who only took drugs before sex (Table 8).

## 4. Discussion

The present study provides new findings about the Chemsex phenomenon in Spanish HIV-infected MSM. The prevalence of Chemsex practice was high between HIV-infected MSM who attended our HIV clinic, taking into account that almost half of the participants (40.6%) in the study had practiced Chemsex at least once during the last year. Engagement in this practice occurred with occasional sexual partners (46.3%), occasional and stable partners (34.2%) and, in a lesser proportion, within stable couples (19.5%). Our results are in accordance with published data from other European countries. In a review of scientific and national surveillance from the United Kingdom, the authors reported a prevalence of 17% in non-HIV MSM attending sexual health clinics and 31% in HIV-infected MSM [17]. The Antiretrovirals, Sexual Transmission Risk and Attitudes (ASTRA) study, which recruited HIV participants aged 18 years or older from eight HIV outpatient clinics, reported a 51% prevalence of Chemsex practices in a sample of 2248 patients [47]. A Spanish study from Madrid in HIV-positive people reported that 29.1% of the sample were Chemsex practitioners [48]. These results show a high prevalence of sexualized drug use in HIV-infected MSM, even higher than in HIV-negative people [17]. Different authors have explored several reasons for Chemsex engagement. Lafortune et al. reported that Chemsex play a role as a coping mechanism that helps individuals to deal with painful emotions or stressful events [49]. Ahmed et al. identified romantic breakups, receiving an HIV diagnosis, the death of a relative, and the accumulation of professional or domestic pressures as triggers of Chemsex practice [50]. In this regard, unpleasant or painful emotional states, such as loneliness [51,52], boredom [51], anxiety [53,54], depression [22], sleep problems [55], stigma associated with HIV-positive status [56], feelings of rejection [57,58] and negative body image [58], are more common among Chemsex users. Another reason why people engage in Chemsex is the perception of lower sexual self-efficacy and sexual pain when using drugs during sex. In fact, it has been found that sexual dysfunction and pain are more prevalent among Chemsex users than non-users [59]. As such, Chemsex practitioners use Chemsex to help themselves feel more attractive and to increase their sexual confidence, pleasure, physical sensations and/or orgasm intensity [57,58]. In this regard, suffering painful emotions, stressful events and psychological problems [60], as well as lower sexual self-efficacy and sexual pain [61], have been previously described as significant predictors of a low HRQoL in PLWHIV. Chemsex could be used as a coping mechanism to deal with distressing emotions such as anxiety, loneliness, boredom, or feelings of rejection, among others [51]. At the same time, this practice could be used to increase self-esteem, emotional closeness and feelings of attractiveness when Chemsex practitioners are struggling with sexual problems [58].

The most common Chemsex drugs used by participants in our study were cannabis, cocaine, methamphetamine, MDMA (Methylene dioxymethamphetamine), GHB (Gamma hydroxybutyrate)/GBL (Gamma butyrolactone), mephedrone, poppers, amphetamines and sildenafil. Cannabis, amphetamines and sildenafil were more frequently used before sex was initiated. This consumption pattern has been reported previously [19]. One of the most commonly used drugs is sildenafil. In general, it is employed by people without sexual problems, such as erectile dysfunction, probably due to the belief that these drugs can increase libido and improve sexual performance, helping to sustain long-lasting sexual activity and reverse the impotence-inducing effects of other substances (e.g., cocaine) or antiretroviral therapy [62]. During sex, participants used cocaine, GHB and poppers more often. GHB is a potent central nervous system depressant and, alongside poppers, can increase the libido, facilitating muscle relaxation to facilitate anal penetration and decrease pain perception [63,64]. Cocaine is probably used to compensate for the depressive symptoms caused by GHB and poppers, thereby increasing stimulation during sex [40]. Methamphetamine, cocaine, MDMA, GHB, ketamine and mephedrone were consumed both before and during sex, probably with the aim of enhancing, disinhibiting or facilitating the sexual experience [17].

Our study shows that Chemsex users have a high risk of transmission of other STDs. In this sense, it has been found that Chemsex practitioners with occasional sexual partners had more condomless sex with partners with discordant or unknown HIV serological status in comparison to non-practitioners. These results are supported by the literature, which has described the relationship between Chemsex practice and a higher number of sexual partners, a higher frequency of condomless sex, and a higher frequency of risky sexual behavior with partners of unknown or HIV-negative status while having a detectable viral load [22,58,65]. In this regard, it has been identified that the use of condoms by many is perceived as a reminder of their HIV status, interfering with their sexual pleasure, while drug use is perceived as a means to achieve a release and to escape from the burdens of HIV stigma [56]. Furthermore, Chemsex practitioners performed higher levels of risky sexual practices with occasional sexual partners, such as double receptive anal penetration, double insertive anal penetration and insertive anal penetration, which puts them at a higher risk of STD transmission [58,65,66,67]. Hence, our results point out the high incidence of STDs in HIV-positive MSM who practice Chemsex. Syphilis, gonorrhea and genital warts were the most frequent STDs in all participants, but only genital warts and urethritis of any origin were significantly more frequent in Chemsex users. In a UK study, authors found similar results in a sample of 1734 participants that used drugs during sex, reporting an increase in the incidence of new diagnoses of HIV infection, acute bacterial Sexually Transmitted Infections (STIs), rectal STIs and hepatitis C [68]. These results highlight the negative consequences of Chemsex on the health of this population.

In this sense, we analyzed the relationship between Chemsex and HRQoL in our sample. Chemsex users obtained lower scores in the domains of General Health Perception, Pain, Energy/Fatigue, Mental Health, Cognitive Functioning, Physical Health Summary and Mental Health Summary. To the best of our knowledge, this is the first study that has analyzed the specific association between Chemsex and HRQoL, employing specific evaluation instruments for the analysis of this health marker in PLWHIV. Moreover, our study points out that those who engage in more extreme forms of Chemsex, i.e., they take drugs both before and during sex, in comparison to those who only take drugs before or during sex, presented lower HRQoL scores, especially regarding Energy/Fatigue and Cognitive Functioning. This is probably related to the fact that a higher frequency of consumption and a larger quantity of drugs consumed could entail a significant deterioration of HRQoL. Taking into account that HIV infection decreases HRQoL [69,70], and also that drug use, such as methamphetamine [71] and recreational cannabis use [72] (among others [73]), affect HRQoL, it seems clear that Chemsex is an important cause of HRQoL decreases in PLWHIV. It is likely that a mixed effect of higher risky sexual practices and drug consumption could be a plausible mechanism to explain the obtained results. However, it is necessary to conduct new studies to identify how Chemsex affects HRQoL.

Although the present study advances our comprehension of Chemsex and its consequences in terms of HRQoL in men with HIV who have sex with men, some limitations should be taken into account. The design of the study only allows us to measure the association of the measures, meaning that we cannot establish any causality. Furthermore, the questionnaire was self-reported by the participants, meaning that some of them may not have been entirely truthful. Moreover, the number of HIV-positive people in the study sample was small, but we were able to confirm our results due to the fact that the participants were representative of MSM in the target population. Finally, the prevalence of depression, which has been previously related to HRQoL impairment, was not evaluated in this study.

## 5. Conclusions

In conclusion, our study highlights the high prevalence of Chemsex in MSM with an HIV infection who are undergoing antiretroviral treatment. MSM with HIV who practice Chemsex participate in more risky sexual practices and make less use of condoms with occasional partners, which leads to a greater risk of STD infection. In this sense, Chemsex is clearly related to a worse HRQoL. Detecting Chemsex practices could provide useful information to clinicians in order to establish prevention and intervention strategies for reducing health deterioration in this population. Future studies are necessary to analyze the specific mechanisms that explain the HRQoL deterioration in MSM who practice Chemsex, and to develop and evaluate the effectiveness of prevention and intervention programs oriented toward a reduction in Chemsex and HRQoL deterioration in MSM with an HIV infection.

## Figures and Tables

**Table 1:** Sociodemographic and serological status of the participants.

		*n* = 101
Age		43.62 ± 11.41
Educational level		
	Primary	11 (10.8%)
	Secondary	16 (15.8%)
	Advanced	38 (37.7%)
	University	36 (35.7%)
Marital status		
	Single	46 (45.5%)
	In a relationship	40 (39.6%)
	Married	10 (9.9%)
	Divorced	4 (4%)
	Widowed	1 (1%)
Employment status		
	Student	3 (3%)
	Employed	76 (75.2%)
	Unemployed	14 (13.8%)
	Pension	8 (8%)
Income level		
	<EUR 1000	19 (18.8%)
	EUR 1001–1500	20 (19.8%)
	EUR 1501–2000	23 (22.8%)
	EUR 2001–2500	16 (15.8%)
	>EUR 2500	23 (22.8%)
Sexual orientation		
	Homosexual	92 (91.1%)
	Bisexual	6 (5.9%)
	Others	3 (3%)
HIV-related variables		
	Time since HIV diagnosis (years)	9.37 ± 6.13
	Current CD4+ lymphocytes (cells/µL.)	812.22 ± 307.33
	Nadir CD4+ lymphocyte (cells/µL.)	469.28 ± 233.62
	Viral load	
	≤50 cop. ARN/mL	93 (92.1%)
	>50 cop. ARN/mL	8 (7.9%)

**Table 2:** Type of drug consumed and time of drug consumption in relation to sex.

	Time of Consumption			
Drug	Never	Yes, Before Sex	Yes, during Sex	Yes, Before and during Sex
LSD (Lysergic acid diethylamide)	41 (100%)	0 (0%)	0 (0%)	0 (0%)
Hallucinogenic Fungus	41 (100%)	0 (0%)	0 (0%)	0 (0%)
Anabolic steroid	41 (100%)	0 (0%)	0 (0%)	0 (0%)
Cannabis	25 (60.9%)	7 (17%)	3 (7.4%)	6 (14.7%)
Cocaine	26 (63.5%)	4 (9.7%)	6 (14.7%)	5 (12.1%)
Crack	41 (100%)	0 (0%)	0 (0%)	0 (0%)
Codeine	40 (97.6%)	0 (0%)	0 (0%)	1 (2.4%)
Methamphetamine	35 (85.3%)	0 (0%)	0 (0%)	6 (14.7%)
MDMA (Methylene dioxymethamphetamine)	32 (78%)	3 (7.4%)	1 (2.4%)	5 (12.2%)
GHB (Gamma hydroxybutyrate)/GBL (Gamma butyrolactone)	30 (73.1%)	2 (4.9%)	5 (12.2%)	4 (9.8%)
Heroin	41 (100%)	0 (0%)	0 (0%)	0 (0%)
Ketamine	39 (95.1%)	0 (0%)	0 (0%)	2 (4.9%)
Khat	40 (97.6%)	1 (2.4%)	0 (0%)	0 (0%)
Mephedrone	35 (85.3%)	1 (2.4%)	2 (5%)	3 (7.3%)
Morphine	41 (100%)	0 (0%)	0 (0%)	0 (0%)
Opium	40 (97.6%)	0 (0%)	0 (0%)	1 (2.4%)
Poppers	23 (56.1%)	2 (4.9%)	12 (29.3%)	4 (9.7%)
Amphetamine	29 (70.7%)	6 (14.7%)	2 (4.9%)	4 (9.7%)
Sildenafil	22 (53.7%)	14 (34.2%)	1 (2.4%)	4 (9.7%)

**Table 3:** Frequency of type of drug consumed.

	Frequency				
Drug	Almost Never	Rarely	Sometimes	Very Often	Always or Almost Always
LSD (Lysergic acid diethylamide)	0 (0%)	0 (0%)	0 (0%)	0 (0%)	0 (0%)
Hallucinogenic Fungus	0 (0%)	0 (0%)	0 (0%)	0 (0%)	0 (0%)
Anabolic steroid	0 (0%)	0 (0%)	0 (0%)	0 (0%)	0 (0%)
Cannabis	0 (0%)	3 (18.8%)	4 (25%)	4 (25%)	5 (31.2%)
Cocaine	1 (6.7%)	8 (53.3%)	5 (33.3%)	0 (0%)	1 (6.7%)
Crack	0 (0%)	0 (0%)	0 (0%)	0 (0%)	0 (0%)
Codeine	1 (100%)	0 (0%)	0 (0%)	0 (0%)	0 (0%)
Methamphetamine	0 (0%)	5 (83.3%)	1 (16.7%)	0 (0%)	0 (0%)
MDMA (Methylene dioxymethamphetamine)	0 (0%)	7 (77.8%)	2 (22.2%)	0 (0%)	0 (0%)
GHB (Gamma hydroxybutyrate)/GBL (Gamma butyrolactone)	0 (0%)	5 (45.4%)	3 (27.3%)	3 (27.3%)	0 (0%)
Heroin	0 (0%)	0 (0%)	0 (0%)	0 (0%)	0 (0%)
Ketamine	0 (0%)	2 (100%)	0 (0%)	0 (0%)	0 (0%)
Khat	1 (100%)	0 (0%)	0 (0%)	0 (0%)	0 (0%)
Mephedrone	0 (0%)	5 (83.3%)	1 (16.7%)	0 (0%)	0 (0%)
Morphine	0 (0%)	0 (0%)	0 (0%)	0 (0%)	0 (0%)
Opium	0 (0%)	1 (100%)	0 (0%)	0 (0%)	0 (0%)
Poppers	3 (16.6%)	5 (27.7%)	7 (38.9%)	1 (5.55%)	2 (11.1%)
Amphetamine	1 (8.3%)	6 (50%)	3 (25%)	1 (8.3%)	1 (8.3%)
Sildenafil	4 (21%)	3 (15.9%)	5 (26.3%)	5 (26.3%)	2 (10.5%)

**Table 4:** Condom use during the last anal penetration with stable and occasional sexual partners.

	Condom Use during the Last Anal Penetration				
	Stable Sexual Partner				
	Total *n* = 59	Chemsex *n* = 25	No Chemsex *n* = 34	χ2	*p*
Yes	24 (40.7%)	8 (32%)	16 (47.1%)	1.354	0.245
No	35 (59.3%)	17 (68%)	18 (52.9%)		
	Occasional sexual partner				
	Total *n* = 68	Chemsex *n* = 33	No Chemsex *n* = 35	χ2	*p*
Yes	55 (80.9%)	23 (69.7%)	32 (91.4%)	5.188	0.023
No	13 (19.1%)	10 (30.3%)	3 (8.6%)		

**Table 5:** Condomless anal intercourse at least once with stable and occasional sexual partners of discordant/unknown serological status.

	Condomless Anal Intercourse at Least Once				
	Stable Sexual Partner of Discordant/Unknown Serological Status				
	Total *n* = 59	Chemsex *n* = 25	No Chemsex *n* = 34	χ2	*p*
Yes	10 (16.9%)	9 (36%)	1 (2.9%)	11.185	0.001
No	49 (83.1%)	16 (64%)	33 (97.1%)		
	Occasional sexual partner of discordant/unknown serological status				
	Total *n* = 68	Chemsex *n* = 33	No Chemsex *n* = 35	χ2	*p*
Yes	18 (26.5%)	11 (33.3%)	7 (20%)	1.551	0.213
No	50 (73.5%)	22 (66.7%)	28 (80%)		

**Table 6:** Prevalence of STD diagnosis for all participants and for practitioners and non-practitioners of Chemsex.

		Total	Chemsex *n = 41*	No Chemsex *n = 60*	χ2	*p*
Genital warts	Yes	27 (%)	16 (39%)	11 (18.3%)	5.324	0.021
	No	74 (%)	25 (61%)	49 (81.7%)		
Genital ulcers	Yes	1 (%)	1 (2.4%)	0 (0%)	1.478	0.224
	No	100 (%)	40 (97.6%)	60 (100%)		
Urethritis	Yes	6 (%)	6 (14.6%)	0 (0%)	9.335	0.002
	No	95 (%)	35 (85.4%)	60 (100%)		
Proctitis	Yes	0 (%)	0 (0%)	0 (0%)	-	-
	No	101 (%)	41 (100%)	60 (100%)		
Syphilis	Yes	41 (%)	21 (51.2%)	20 (33.3%)	3.231	0.072
	No	60 (%)	20 (48.8%)	40 (66.7%)		
Chlamydia	Yes	11 (%)	6 (14.6%)	5 (8.3%)	0.996	0.318
	No	90 (%)	35 (85.4%)	55 (91.7%)		
Candidiasis	Yes	6 (%)	4 (9.8%)	2 (3.3%)	1.798	0.180
	No	95 (%)	37 (90.2%)	58 (96.7%)		
Gonorrhea	Yes	25 (%)	12 (29.3%)	13 (21.7%)	0.756	0.385
	No	76 (%)	29 (70.7%)	47 (78.3%)		

**Table 7:** Differences in Health-Related Quality of Life (HRQoL) between participants who practiced Chemsex and those who did not.

	Chemsex *n* = 41	No Chemsex *n* = 60	t	*p*	Effect Size
General Health Perception	56.09 ± 24.14	68.75 ± 20.98	t(99) = 2.798	0.006	*d* = 0.56
Pain	65.85 ± 24.01	77.96 ± 21.88	t (99) = 2.625	0.010	*d* = 0.52
Physical Functioning	87.60 ± 17.88	91.38 ± 13.54	t (99) = 1.210	0.229	*d* = 0.24
Role Functioning	96.34 ± 17.28	97.50 ± 34.96	t (99) = 0.196	0.845	*d* = 0.03
Social Functioning	84.87 ± 23.57	91.33 ± 17.41	t (68.905) = 1.497	0.139	*d* = 0.36
Energy Fatigue	63.53 ± 19.50	74 ± 15.88	t (99) = 2.961	0.004	*d* = 0.59
Mental Health	64 ± 19.73	71.73 ± 16.43	t (99) = 2.139	0.035	*d* = 0.42
Health Distress	78.65 ± 26.29	87.91 ± 16.52	t (61.477) = 2.001	0.050	*d* = 0.51
Cognitive Functioning	74.51 ± 21.47	84.25 ± 14.01	t (99) = 2.759	0.007	*d* = 0.55
Quality of Life	62.19 ± 21.73	68.75 ± 17.60	t (99) = 1.669	0.098	*d* = 0.33
Health Transition	59.75 ± 24.92	60.41 ± 17.40	t (66.112) = 0.147	0.884	*d* = 0.03
Physical Health Summary	68.43 ± 16.85	78.01 ± 13.58	t (99) = 3.153	0.002	*d* = 0.63
Mental Health Summary	69.47 ± 18.36	78.55 ± 12.34	t (64.343) = 2.769	0.007	*d* = 0.69

**Table 8:** Differences in HRQoL between participants who took drugs before sex, during sex or before and during sex.

	Chemsex *n* = 41				
	Before Sex *n* = 10	during Sex *n* = 13	Before and during Sex *n* = 18		
	Mean rank	Mean rank	Mean rank	H _(df)_	*p*
General Health Perception	25.80	21.96	17.64	3.125 _(2)_	0.210
Pain	26.80	18.42	19.64	3.261 _(2)_	0.196
Physical Functioning	24.70	20.27	19.47	1.480 _(2)_	0.477
Role Functioning	22	18.85	22	4.415 _(2)_	0.110
Social Functioning	26.95	20.81	17.83	5.037 _(2)_	0.081
Energy Fatigue	29.20	17.23	19.17	6.459 _(2)_	0.040
Mental Health	28.55	19.08	18.19	5.322 _(2)_	0.070
Health Distress	25.75	20.77	18.53	2.448 _(2)_	0.294
Cognitive Functioning	30.55	21.46	15.36	10.478 _(2)_	0.005
Quality of Life	26.65	21.65	17.39	4.472 _(2)_	0.107
Health Transition	22.90	21.77	19.39	0.756 _(2)_	0.685
Physical Health Summary	28.60	19.73	17.69	5.548 _(2)_	0.062
Mental Health Summary	29.80	19.46	17.22	7.412 _(2)_	0.025

df (degrees of freedom).

## Data Availability

The data are not publicly available due to reasons concerning privacy of the subjects and since it belongs to an ongoing project.
